# Learn to optimize—a brief overview

**DOI:** 10.1093/nsr/nwae132

**Published:** 2024-04-02

**Authors:** Ke Tang, Xin Yao

**Affiliations:** Department of Computer Science and Engineering, Southern University of Science and Technology, Shenzhen 518055, China; Department of Computing and Decision Sciences, Lingnan University, Hong Kong 999077, China

**Keywords:** optimization, data-driven algorithm design, automated algorithm configuration, machine learning

## Abstract

Most optimization problems of practical significance are typically solved by highly configurable parameterized algorithms. To achieve the best performance on a problem instance, a trial-and-error configuration process is required, which is very costly and even prohibitive for problems that are already computationally intensive, e.g. optimization problems associated with machine learning tasks. In the past decades, many studies have been conducted to accelerate the tedious configuration process by learning from a set of training instances. This article refers to these studies as *learn to optimize* and reviews the progress achieved.

## Teaser

The article presents an overview on “Learn to Optimiz”, a paradigm that leverage on a set of training instances to accelerate the tedious configuration process of optimization algorithms.

## INTRODUCTION

The great success achieved by machine learning (ML) is backed up by the development and application of optimization techniques. For example, most supervised learning tasks require solving the optimization problem


(1) \begin{eqnarray*}
\min J = \int _\mathbf {x}l(h(\mathbf {w}, \mathbf {x}), y)\mathrm{d}\mathbf {x},
\end{eqnarray*}

where *l* is the loss function, **x** is the training data and *y* is the label of training data. The parameter(s) **w** of the learning machine *h*, e.g. the weights of a neural network (NN), is (are) trained by minimizing the objective function in Equation (1). ML tasks may involve various loss functions and representations of learning machines, which lead to objective functions that favor different optimization methods. As a result, many optimization techniques, including convex optimization methods [1], gradient descent [2], heuristic search [3] as well as evolutionary algorithms (EAs) [4], have been utilized by the ML community in the past decades.

Mainstream optimization algorithms are usually highly configurable parameterized algorithms, i.e. they involve tunable parameters. Performance of such algorithms may vary significantly with different parameter settings. Hence, to solve the optimization problem induced by a learning task, tedious efforts are typically needed to configure the optimization algorithm. Consider the case of training an NN with stochastic gradient descent (SGD). It is widely acknowledged that the training performance is sensitive to the learning rate [5], a parameter of SGD. How to determine the optimal or at least appropriate learning rate has become a major hurdle for training NNs, especially for large-scale NNs [6], or when the objective function is not an explicit differentiable function of the weights of an NN, as in the typical reinforcement learning setting [7].

**Figure 1:**
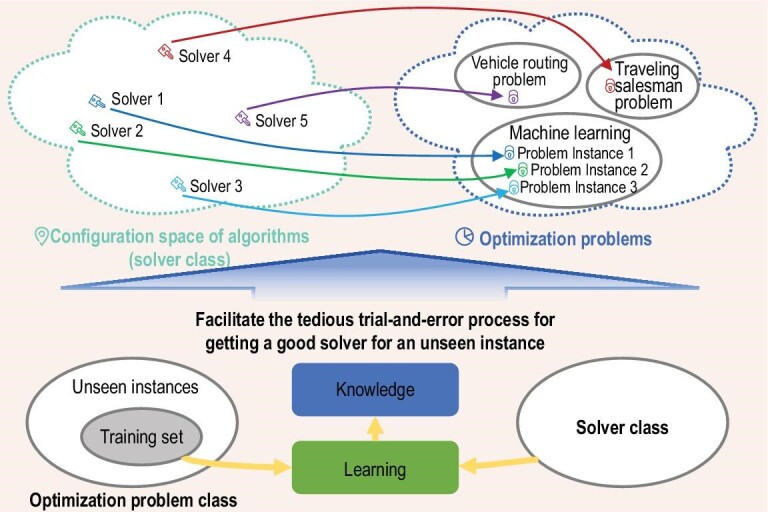
Illustration of the general idea of L2O.

In order to reduce the human labor required, many studies have attempted to automate the configuration of optimization algorithms [6,8]. However, such approaches are, in many cases, still computationally prohibitive since at least dozens of candidate parameter settings need to be tried, making the configuration process much more time consuming than the training process itself [9]. Hence, how to further alleviate such a cost is attracting more and more attention in the ML community [10]. Interestingly, taking a look into the literature of optimization, we find that many more investigations have been performed along a similar direction since the 1990s or even earlier, published under different names, such as algorithm selection [11], algorithm configuration [8] and algorithm design [12]. These studies share the same general paradigm that is very similar to the typical ML paradigm. That is, they leverage on a set of training instances from the target optimization problem class to gain something that would help alleviate the tedious algorithm configuration process on unseen problem instances, as illustrated in Fig. 1. This article puts them under the umbrella term *learn to optimize* (L2O) and provides a brief overview on the general ideas as well as critical issues of L2O, including the training process, theoretical foundations as well as generalization issues. For more algorithmic details on NN-based solvers and heuristic solvers, readers are referred to [13,14] and [15,16], respectively.

## PRELIMINARIES

A concrete case of L2O depends on two issues: the target optimization problem class and the configuration space of optimization algorithms. For the sake of clarity, we henceforth refer to the former as the target problem class and the latter as the solver class.

A problem class is a set of problem instances that share some common properties, and its definition could be very flexible. For example, the simplest objective function for supervised learning as in Equation (1) defines a problem class. An instance of this problem class is the most concrete optimization problem with all details of Equation (1) specified, including the form of the loss function, the representation of the learning machine and the training data. Alternatively, one could also define the loss function as the mean squared error and the learning machine to be an NN; instances of the corresponding problem class are then defined over different training datasets.

The solver class defines the algorithm class under consideration. Each element of the solver class is a solver, which is an algorithm with all parameters assigned with concrete values. An intuitive example of the solver class is defined over the learning rate of SGD. In this case, each unique value for the learning rate corresponds to a solver. Alternatively, a solver could also be defined by a learning rate schedule or an adaptive scheme like Adam [17]; the solver class then corresponds to a set of learning rate schedules and adaptation functions, respectively. It should be noted that a solver class could be defined in many (almost arbitrary) forms, albeit parameters of an optimization algorithm might be the most intuitive ones. For instance, the configuration space could be simply a set of strings since any algorithm is implemented as codes [18], or a set of heuristic rules/functions for heuristic search frameworks like A* [19] or EAs [20].

Given a target problem class **I**, a training instance set **I**_*tr*_ and a solver class **A**, L2O aims to establish an efficient way to automatically find a good solver for the problem instances in **I**∖**I**_*tr*_. In addition to such a definition, two issues are worthy of further discussion. First, L2O is relevant to several topics in ML and optimization. From the ML perspective, L2O is closely related to so-called meta-learning [21] as both concern multiple optimization/learning tasks and we tackle them through thinking at a higher abstract level. But the two research topics differ in two aspects. On the one hand, L2O is not restricted to a special optimization problem class, while meta-learning mainly concerns learning problems. On the other hand, L2O mainly concerns finding a good solver, while manipulation of the training data [22] and structure of learning machines [23] is also taken into account in meta-learning.

From the viewpoint of optimization, ML ideas have been introduced into optimization for a long time. Dating back to the early literature on the self-adaptive optimization algorithm, ML techniques have been used to adapt parameters of an optimization algorithm on the fly [24]. In this setting, all data used for learning are acquired from the solving process of a single problem instance, and the knowledge learned will only be used for this problem instance. Multi-task optimization [25], on the other hand, assumes that multiple problem instances are to be solved in parallel and aims to solve each problem instance better (or more efficiently). These studies are essentially different from L2O because they only care about the performance on the given problem instance(s) and generalization to unseen problem instances is not emphasized.

Second, if there exists a solver that performs the best on all instances of the target problem class, L2O is clearly expected to identify this solver from the solver class. However, for an optimization problem class of practical significance, it is unlikely that a universally best algorithm instance exists [26,27]. This is exactly the motivation for configuring an algorithm for each problem instance. Since L2O tries to avoid carrying out the tedious trial-and-error configuration process for each problem instance, one may not expect it to lead to the best performance on all instances. Instead, it is more appropriate to view L2O as an attempt to find a better trade-off between the algorithm configuration cost and the optimization performance (e.g. in terms of runtime, solution quality, etc.).

**Figure 2:**
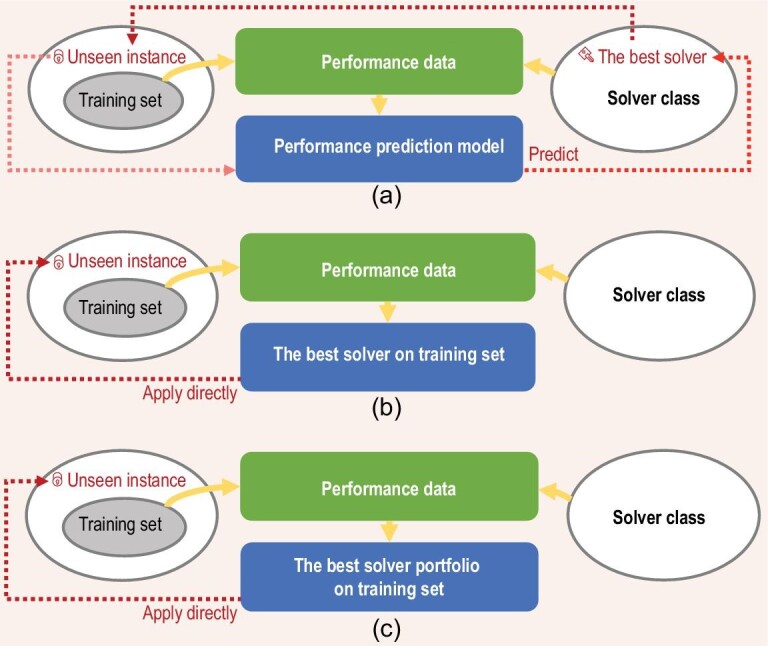
Illustration of three training methods of L2O: (a) training performance prediction models, (b) training a single solver and (c) training a portfolio of solvers.

A first question about L2O is what is to be obtained through the training process. Previous studies can be categorized into three groups: training a solver performance prediction model, training a single solver and training a portfolio of solvers; see Fig. 2.

### Training performance prediction models

Given a solver and training instances from the target problem class, a performance prediction model is typically trained in the following way [28]. First, each training instance is described by a set of features. These features are often manually designed to extract the characteristics for the given problem instances and should be efficiently computable [11], typically in low-order polynomial time concerning the size of the given problem instance. At the same time, performance data are gathered by running the solver on each training instance. Here, the performance measure can be defined in various forms [29], such as runtime, solution quality, memory usage, energy consumption and communication overhead. The performance data are then utilized as labels and standard ML techniques could be used learn a mapping from instance features to the performance of the solver.

The core idea of the approach described above, i.e. establishing a mapping from instance features to the performance of a solver, first appeared in the 1970s [11]. After that, there has been growing research interest in applying this idea to different ML problems [30] such as classification [31], regression [32] and time-series forecasting [33]. Beyond ML, the idea of training solver performance models was later introduced into optimization and decision-making domains, specifically the Boolean satisfiability problems (SATs) and combinatorial auction winner determination problems, and are referred to as empirical hardness models [27] and empirical performance models [29].

The idea of training a performance prediction model has been naturally extended in several ways. Leyton-Brown *et al.* [27] proposed including solver parameters as the inputs of the performance prediction model, enabling the model to predict the performance of any given solver on any given problem instance. Another extension involves predicting not only the performance on a single instance, but also the average performance on the training set [29]. Subsequently, building solver performance prediction models has been widely applied to various optimization problems such as the traveling salesman problems (TSPs) [34,35], vehicle routing problems (VRPs) [36,37] and quadratic assignment problems [38,39]. Research along this line often concerns a particular problem class of interest and revolves around (1) designing problem features or uncovering characteristics that are likely to correlate with the performance of solvers, (2) building better models to capture the relationship between problem characteristics and the performance of solvers. More recently, researchers have started to explore the development of cross-domain performance prediction models [40], meaning that the features are no longer problem specific but rather domain agnostic.

Creating performance prediction models directly makes it possible to select or recommend the best solver for a problem instance with little effort, referred to as automatic algorithm selection (AAS)[15]. Suppose that a performance prediction model has been trained. Given an unseen problem instance to solve, AAS adopts the performance prediction model to choose the best solver from a finite solver class. According to the type of performance prediction model, AAS can be implemented via at least two different strategies [28]: regression and classification. The former explicitly constructs a mapping between instance features and the performance of candidate solvers. Performance of each candidate solver on the unseen problem instance is estimated by the performance prediction model, and the solver with the best estimated performance is selected. In comparison, the classification strategy transforms performance data into (instance, label) pairs, where the label represents the best-performing solver on that instance. In this way, a classification model is built to directly predict the best algorithm for a given problem instance. Besides, alternative strategies, such as predicting rankings of solvers [41] and then selecting the top-ranked algorithm, have also been explored.

AAS has found success in various optimization problem domains, including TSPs [34], knapsack problems [42], bin-packing problems [43] and continuous black-box optimization problems [44,45]. However, its wider application has encountered non-trivial obstacles. Specifically, the accuracy of a performance model heavily depends on informative problem features, which are typically designed by domain experts. Over the years, many problem domains (such as TSP) have accumulated their own set of features, yet at the core they are mostly a collection of everything that was considered useful in the past [34,35]. This implies that labor-intensive feature engineering is often needed to identify useful features. Furthermore, for a new problem class, new features are needed, while designing them is non-trivial and requires significant domain expertise and human efforts [29,46]. To overcome these hurdles, some recent works [35,47–49] explored the use of deep learning for feature-free algorithm selection. By directly encoding problem instances into a latent representation, the problem features and the performance prediction model are learned simultaneously. Seemingly promising, such end-to-end AAS methods still need to be validated on more problem classes and more comprehensively.

### Training a single solver

The performance prediction model set up a nice interface for utilizing various ML techniques to better solve optimization problems. However, the accuracy of the performance prediction model strongly relies on the amount of training data, while getting a single datum requires running a solver on a problem instance, which involves a non-trivial computational cost. For a fixed budget of training data, it could be expected that the accuracy of the performance prediction model decreases rapidly with the cardinality of the solver class (i.e. how many solvers are considered in the class). Therefore, the performance prediction model approach is typically restricted to cases with a finite solver class, and, desirably, of relatively small size.

If the solver class is defined over continuous parameters of an optimization algorithm then the solver class will be an infinite set. Most AAS methods may not be directly applied to such an infinite solver class. Instead, it is more common to search for the best solver in the solver class, termed the automatic algorithm configuration (AAC) in the literature [16]. More specifically, given a solver class, the solver that achieves the best overall performance on the training instances is first obtained by some search method. Then, the solver is directly applied to an unseen problem instance without a further configuration process.

A typical AAC method consists of two main components: sampling in the solver class and evaluating a candidate solver. Sampling in the solver class is mostly implemented based on search-based methods and model-based methods. Search-based methods such as hill climbing [50], local search [8] and EAs [51] have been used to sample solvers, since they make minimal assumptions about the solver representation and do not require gradients. Additionally, some researchers have also explored using experimental design [52,53], such as the full factorial experimental design, to generate the starting points for the search. In comparison, model-based methods are more similar to the performance prediction model approach. They seek to capture the dependencies between solvers and their performance, by training surrogate models on the performance data collected during the configuration process. On the one hand, the surrogate model is used to guide the search process. On the other hand, the performance data of the sampled solver are used to refine the surrogate model to enhance its prediction accuracy. Commonly used model-based methods include estimation of distribution algorithms [54,55] and Bayesian optimization algorithms [56].

The solver evaluation process becomes an issue because a solver may behave differently over training instances for many problem classes, e.g. NP-hard problems. Suppose that a set of training instances from TSPs and the evaluation of a solver involves a total *N* runs of the solver on the training instances. For some instances, the performance of the solver might be very stable and running it once would be sufficient. But, for the other instances, the solver might not be that stable and multiple runs would be needed to obtain a reliable estimate of the solver’s performance. A research question then arises of how to allocate the *N* runs to the training instances to make a most proper evaluation of the solver on the whole training set. Research on this question is relatively limited. A recent work [57] proved that a universal evaluator, which evenly distributes the *N* runs across all training problem instances, can maximize the reliability of the evaluation result (somewhat counterintuitive). Some other studies on more specific problem classes [58–60] showed that tailored evaluation strategies, rather than the uniform allocation strategy, can lead to faster convergence of the search process when the performance of the solver follows a heavy-tailed distribution.

Based on various search/sampling methods and evaluation strategies, quite a few concrete AAC methods have emerged in the past decades [8,51,55,56]. These methods are capable of handling various types of parameters (e.g. numerical, integer and categorical), high-dimensional parameter spaces (e.g. number of parameters >100) and different performance metrics. Successful case studies have been reported on various problem domains, including SAT [51], TSP, the mixed integer programming problem [61] and VRPs [62].

Most studies on AAC consider parameterized heuristic algorithms as their solver class. Recently, inspired by the success of deep reinforcement learning (DRL) [63], there has been increasing research interest in utilizing DRL to train a deep NN as a component of the solver for combinatorial optimization problems. The resultant paradigm is usually termed neural combinatorial optimization (NCO) [64]. Existing NCO methods are categorized into learning constructive heuristics (LCH) [64,65] and learning improvement heuristics (LIH) [66]. As the names suggest, the solvers trained by LCH methods and LIH methods are constructive heuristics and improvement heuristics, respectively. Taking the TSP as an example, the conventional greedy heuristic for TSPs always selects the closest city for insertion. In comparison, LCH approaches learn an NN to score each city and select the one with the highest score for insertion [64]. LIH approaches, instead, learn an NN to select which parts of the TSP solution are to be improved and how to improve them (e.g. selecting which cities to exchange positions) [66]. Though several recent experimental studies [67,68] reported that the performance of NCO approaches remains somewhat unsatisfactory, research in this area is still in fast development, continuously absorbing ideas from both deep learning and traditional combinatorial optimization. More recently, attempts to apply similar ideas to other types of optimization problems, such as continuous optimization and multi-objective optimization, have also appeared in the literature [69,70].

### Training a portfolio of solvers

By considering more complicated solver structures, recent research evolved into the realm of training algorithm portfolios (APs)[71]. That is, a portfolio of solvers, rather than a single solver, is obtained from the solver class. Both AAS and AAC methods could be directly applied in this case. However, the extension from training a single solver to training a portfolio of solvers inherently introduces a higher degree of freedom. For example, suppose that the cardinality of the solver class is *n*, and that the AP consists of *k* solvers. The number of candidate APs is $C_n^k$ and increases fast with *k*. This makes training an AP much more challenging than training a single solver, and thus motivated quite a few studies on specified methods for training APs. For example, Hydra [72] constructs an AP by iteratively finding a solver that maximizes the marginal performance contribution to the current AP. The instance-specific algorithm configuration (ISAC) [73] first clusters the training problem instances into groups and independently identifies a solver for each cluster. Parallel configuration with instance transfer (PCIT) [74] also adopts an instance grouping strategy, but dynamically transfers instances between groups during the training process.

Another interesting research question arising with AP is how to utilize the obtained AP for solving unseen problem instances. The most naïve way is to run all solvers in the portfolio in parallel [75]. In this case, it is not surprising that AP would lead to better solution quality [76,77], but with a higher computational cost (than running a single solver) as the payoff. Alternatively, one can also treat the AP as a smaller solver class than the original one and select a single best algorithm to solve a new problem instance [72,73]. In this case, training an AP becomes a ‘pre-processing’ stage aiming to reduce the size of the solver class for AAS. Some studies also considered running each solver in the AP sequentially with pre-allocated time budgets [78]. It should be noted that the objective function for training APs not only depends on the performance measure of the individual solvers in the AP, but also on the way in which the AP is utilized. Hence, the above-mentioned training methods, such as Hydra, ISAC and PCIT, might need to be adapted according to the way in which APs are utilized; see, e.g. [79].

### Theoretical guarantee for training

The training methods discussed above have achieved much practical success, but they rely on many heuristics and lack a theoretical guarantee. In particular, since any L2O approach involves a higher-level optimization problem, the most obvious theoretical question is how much time is required (or what is the most efficient way) to get the optimal (or near-optimal) solver given the training instances. This question has been touched on in the past few years and preliminary results independent of the target problem class have been obtained; see [58–60,80] for examples. However, these theoretical results were mainly empirically validated on very classical problem classes such as SAT [59,60]. Thus, their practical significance might be clearer in the years to come.

## GENERALIZATION ISSUES

Generalization is one of the most critical issues for ML. The generalization issue in the context of L2O means how well the learned solver would perform on unseen instances of the target problem class. Compared to the relatively more mature studies on the training phase of L2O, answers to two research questions regarding generalization remain unclear.

The first question is how to measure, either theoretically or empirically, the generalization performance of a learned solver. In the traditional literature of optimization, generalization of a solver is typically stated as ‘an algorithm has an approximation ratio of *a* and time complexity of *b* for the problem class *c*’. Such a statement, although it seems quite different from what one may see in a typical ML paper, inherently says that the approximation ratio and time complexity hold for all instances of the target problem class and thus provide a rigorous guarantee for the generalization performance. However, in the context of L2O, it will be much more challenging to obtain such a strong statement for two reasons. First, a major potential drawback of L2O is that the problem classes are too complex for humans to design an algorithm that has both a desirable performance and rigorous performance guarantee. In this case, it might be unrealistic to expect a machine to achieve such an accomplishment. Second, the representation of the solver may also bring new challenges. Suppose that an NN is to be learned as a solver; it would not be surprising that generalization of the learned NN can hardly be rigorously analyzed.

Having said the above, progress has been made along this direction. For example, Gupta and Roughgarden [81] introduced the probably approximate correct learning framework into the L2O context, and proved that a parameterized greedy algorithm with generalization bound could be learned for the knapsack and maximum weight independent set problems. More recently, a general analytical approach has been derived for solver classes with continuous tunable parameters [82], which shows that a generalization bound (specifically, the difference between the solver’s average performance over the training set and its expected performance on the target problem class) holds with probability 1 − δ if the target problem class and the solver class fulfill certain conditions. With the aid of this approach, more case-by-case results have been obtained on well-known solver classes, such as integer programming and integer quadratic programming algorithms [83], and on more problem classes such as sequence alignment [82] and clustering [83].

Since it is very hard to theoretically analyze the generalization of a learned solver, most studies on L2O so far resort to the common practice in ML, i.e. using a separate testing instance set to empirically estimate the generalization. However, such a protocol works only if the training and testing instances are drawn from the same distribution (or at least similar distributions). For many problem classes, such as combinatorial optimization problems, it is even non-trivial to measure the similarity between two instances. As a result, one may have to either adopt some ad hoc rules to split the problem instances into training or testing sets [74], or identify some problem features to first map the problem instances into a Euclidean space [84], in which instance splitting could be done more easily. The former would provide very limited evidence for the true generalization performance since the link between the ad hoc rules and the distribution of unseen instances are largely unclear. The latter heavily relies on human knowledge to design the problem features, and thereby contradicts the motivation of L2O.

The second research question, which might be more important than measuring the generalization performance, is how to improve it. Unfortunately, very few studies have been dedicated to this sub-topic. Two general ideas could be borrowed from the ML literature. The first idea is to introduce some type of regularization into the training process, e.g. introducing a regularization term that controls the complexity of the learning machine. However, if the learning machine becomes a solver, it would be very difficult to design such a regularization term. On comparison, the other idea, which is to generate pseudo instances to form a training set that better represents the target problem class, seems to be more tractable. Suppose that an instance generator is available; the question then becomes how to generate pseudo instances that, if inserted into the training set, would improve the generalization of the learned solver. Suppose that a solver has been trained and performs well on all current training instances. A useful new training instance would be the one that is hard (i.e. cannot be solved well) for the current solver. Motivated by this intuition, a generative adversarial approach has been proposed [85,86]. The idea is to alternately train the solver and generate new pseudo instances that are hard for the current solver. In essence, this approach could be viewed as greedily maximizing a generalization bound of the learned solver and is not restricted to any specific problem class. Case studies on training APs for SAT [85], TSP [85,86] and a more realistic vehicle routing problem with simultaneous pickup delivery and time windows [86] all show that such an approach leads to APs that generalize better.

## CONCLUSIONS AND DISCUSSIONS

For optimization problems of broad interest (e.g. NN training), state-of-the-art solvers are usually highly configurable. Performance of these solvers, typically in terms of runtime or solution quality, are volatile functions of many design choices such as the type of solver (SGD or EA), search operators and the values of the solvers’ parameters. Given a target problem class, determining these design choices for each problem instance would be too costly in terms of either human labor or computational cost. It is thus desirable if a machine is able to learn to identify the most appropriate solver from a solver class. Under the umbrella term L2O, this article reviews the efforts dedicated to this purpose.

From this article, it can be found that fruitful approaches have been developed for L2O. Most of them are general-purpose approaches, i.e. they were not developed for a specific problem class. However, L2O has been shown to achieve human-competitive (or even better) performance not only on traditional optimization problems, but also on recently emerging problems, such as pose estimation [87], adversarial attack [76] and chip placement [88]. Because of the ever-increasing cost for manually designing/configuring a solver for ML, L2O is expected to grow into a critical technology that eases the increasingly unaffordable human labor in this context.

Finally, the difficulty in measuring and improving generalization performance makes it very challenging for L2O to warrant reasonable generalization, especially when the complex problem class and solver class are considered. Thus, it might be unreliable to directly apply the learned solvers on unseen problem instances. We thus hypothesize that a second-stage fine-tuning might be necessary in many real-world scenarios. If this is the case, the learned solver(s) could be viewed as foundation models for further fine-tuning. In fact, training an AP could be viewed as a special case of such an idea. To be specific, if a solver is to be selected from the learned AP for each unseen problem instance then the solver selection procedure is the simplest way of fine-tuning. Alternatively, one may also use the learned solver(s) as starting points and implement more complex fine-tuning procedures, e.g. introducing a round of search (hopefully less costly), or transferring the learned solvers to unseen instances [89]. Consequently, how to build up a synergy between the training and fine-tuning of foundation models would be a critical direction to fully deliver the potential of L2O in AutoML, and even for optimization in general.
